# Multiplication of *ampC* upon Exposure to a Beta-Lactam Antibiotic Results in a Transferable Transposon in *Escherichia coli*

**DOI:** 10.3390/ijms22179230

**Published:** 2021-08-26

**Authors:** Tania S. Darphorn, Yuanqing Hu, Belinda B. Koenders-van Sintanneland, Stanley Brul, Benno H. ter Kuile

**Affiliations:** 1 Laboratory for Molecular Biology and Microbial Food Safety, Swammerdam Institute for Life Sciences, University of Amsterdam, Science Park 904, 1098 XH Amsterdam, The Netherlands; T.S.Darphorn@uva.nl (T.S.D.); hyq1708@mnnu.edu.cn (Y.H.); b.b.koenders-vansintanneland@uva.nl (B.B.K.-v.S.); s.brul@uva.nl (S.B.); 2 School of Biological Science and Biotechnology, Minnan Normal University, Zhangzhou 363000, China; 3 The Netherlands Food and Consumer Product Safety Authority, Office for Risk Assessment, Catharijnesingel 59, 3511 GG Utrecht, The Netherlands

**Keywords:** beta-lactam resistance, transposon, plasmid, horizontal gene transfer

## Abstract

Plasmids play a crucial role in spreading antimicrobial resistance genes. Plasmids have many ways to incorporate various genes. By inducing amoxicillin resistance in *Escherichia coli*, followed by horizontal gene transfer experiments and sequencing, we show that the chromosomal beta-lactamase gene *ampC* is multiplied and results in an 8–13 kb contig. This contig is comparable to a transposon, showing similarities to variable regions found in environmental plasmids, and can be transferred between *E. coli* cells. As in eight out of nine replicate strains an almost completely identical transposon was isolated, we conclude that this process is under strict control by the cell. The single transposon that differed was shortened at both ends, but otherwise identical. The outcome of this study indicates that as a result of exposure to beta-lactam antibiotics, *E. coli* can form a transposon containing *ampC* that can subsequently be integrated into plasmids or genomes. This observation offers an explanation for the large diversity of genes in plasmids found in nature and proposes mechanisms by which the dynamics of plasmids are maintained.

## 1. Introduction

Plasmids have a crucial role in spreading genes coding for antimicrobial resistance, and a large variety of resistance plasmids has been described in considerable detail [1,2]. In contrast, the ontogeny of resistance plasmids is not well understood. What are the driving factors causing the genesis of plasmids and their subsequent spreading? That a microbe carrying a plasmid coding for resistance against a certain antibiotic will have a selective advantage when exposed to that compound is obvious, but where did that plasmid originate from in the first place? The chromosomal *ampC* gene of *Escherichia coli* codes for a beta-lactamase but is normally expressed at very low levels. Mutations in the promoter region induced by exposure to beta-lactam antibiotics cause overexpression, which results in resistance to certain beta-lactam antibiotics, such as amoxicillin. This gene is multiplied between 20 to 600 times when *E. coli* is made resistant to amoxicillin by exposure to stepwise increasing concentrations [3]. This study addresses the question whether the multiple copies of the *ampC* gene can form a transposon that could possibly be transferred to other *E. coli* cells. 

An *ampC* gene is present in the *E. coli* chromosome but can also be carried in plasmids under several different names, depending for example on the bacterial species they originate from or on additional resistance gained through small mutations, such as the *CMY* gene [4]. The *ampC* gene enables resistance against several beta-lactam antimicrobials [5]. In many *Enterobacteriaceae*, including *E. coli*, the *ampC* gene does not result in a high level of resistance until mutations and genomic changes occur after exposure to antibiotics [3,6]. Different kinds of mutations have been described, mostly in the promoter/attenuator region [7,8,9,10,11,12,13] or in inhibitory genes of *ampC*, such as *ampR* [14]. In addition, multiplication of the gene has been observed [3]. In the end, all these mutations result in high levels of beta-lactam resistance. Similar to multiplication, transducing phages cause high resistance levels to beta-lactams by multiplying *ampC* [15]. Within plasmids, higher copy numbers of the gene also increase resistance, but this is less effective in increasing resistance than genetic reorganization, promoter mutations, and modifying the overall genetic environment [16]. Still, for strains that do not contain any plasmids, amplifying *ampC* seems to be a viable strategy for survival when exposed to beta-lactam antibiotics [3,17]. 

The fate of the multiplied *ampC* is not known. Hoeksema et al. (2018) [3] showed that the *ampC* gene is multiplied within larger fragments of 3–10 kbp that originated from the same position in the genome of *E. coli*. Whether this fragment becomes an extra-chromosomal structure and if it is stable as such, is unknown. Similarities between the fragments found and clusters present in sequenced plasmids [6,18] give rise to the question whether this multiplication might be a possible intermediate step in the transfer of *ampC* from the genome in *E. coli* into plasmids. To understand these processes, this study amplified *ampC* by exposing *E. coli* to stepwise increasing concentrations of amoxicillin. Plasmid isolation and sequencing were used to determine the exact composition. Transfer experiments verified whether the fragments’ DNA could spread from a newly resistant cell to a susceptible cell and whether it caused resistance in the new host.

## 2. Results

**Figure 1:**
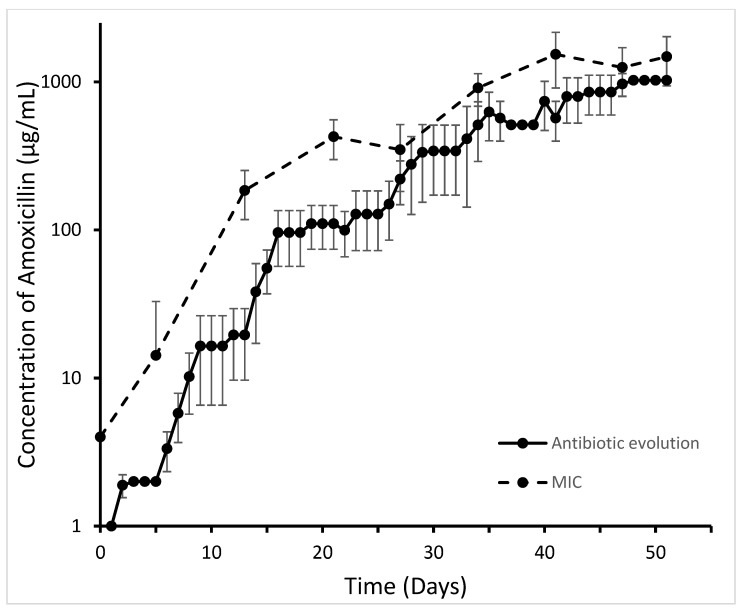
Evolution of amoxicillin resistance of nine *E. coli* MG1655 strains exposed to stepwise increasing concentrations of antibiotic (antibiotic evolution) and the corresponding MIC for amoxicillin (MIC).

To examine the hypothesis that *E. coli* MG1655 can form a transferable DNA fragment upon induction of de novo beta-lactam resistance, first of all, nine replicate strains of wild-type MG1655 were made resistant to amoxicillin. During the first 15 days of exposure to stepwise increasing concentrations of amoxicillin, resistance rapidly increased, followed by 35 days of slower increase to a final value of 1024 µg/mL (Figure 1). The minimal inhibitory concentrations (MIC) measured by a standard methodology were always slightly higher than the highest concentrations that still allowed growth in the experimental setup. In control experiments, amoxicillin resistance remained at 4 µg/mL. The final MIC for ampicillin, an antibiotic closely resembling amoxicillin, was comparable at 1024 µg/mL, and those for chloramphenicol and tetracycline remained at wild-type levels, i.e., 16 and 4 µg/mL, respectively. 

To determine whether the de novo acquired resistance was permanent or temporary, at the end of the experiment, three amoxicillin-resistant replicates were transferred daily without antibiotics, three in the presence of 4 µg/mL of amoxicillin, and three with 1024 µg/mL of amoxicillin added to the medium. In all cases, the MIC remained unchanged at 1024 µg/mL for 14 days, which corresponds to approximately 100 generations.

### 2.1. Transposon Containing ampC

**Figure 2:**
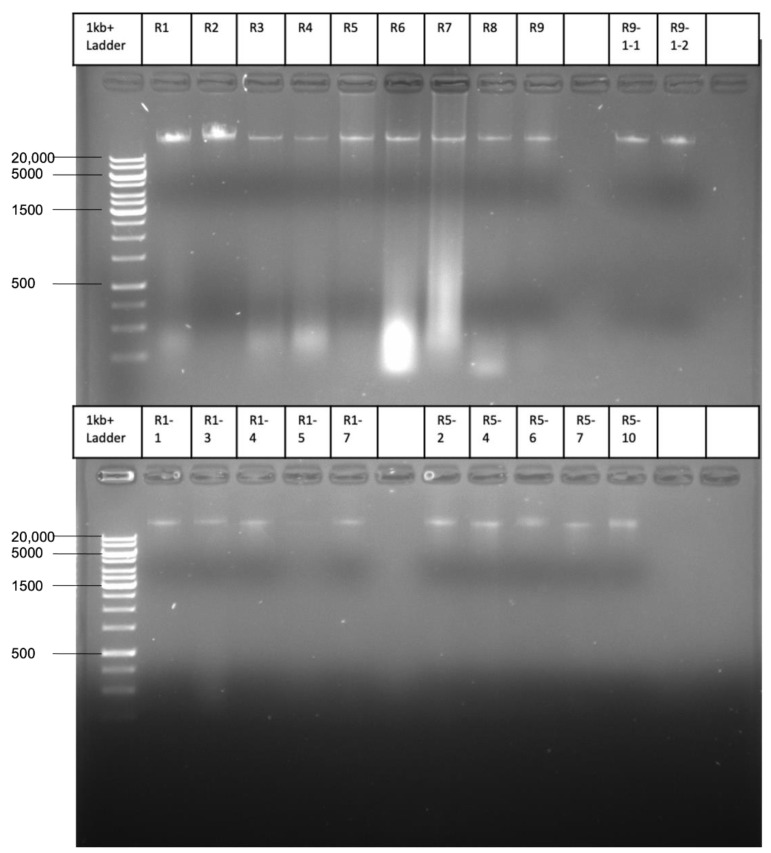
Gel after DNA isolation with the Qiagen plasmid maxi kit of the endpoints of evolution from the nine replicates (R1–R9, with MIC for amoxicillin around 1024 µg/mL) and the expected transformants for R9, R1, and R5.

**Figure 3:**
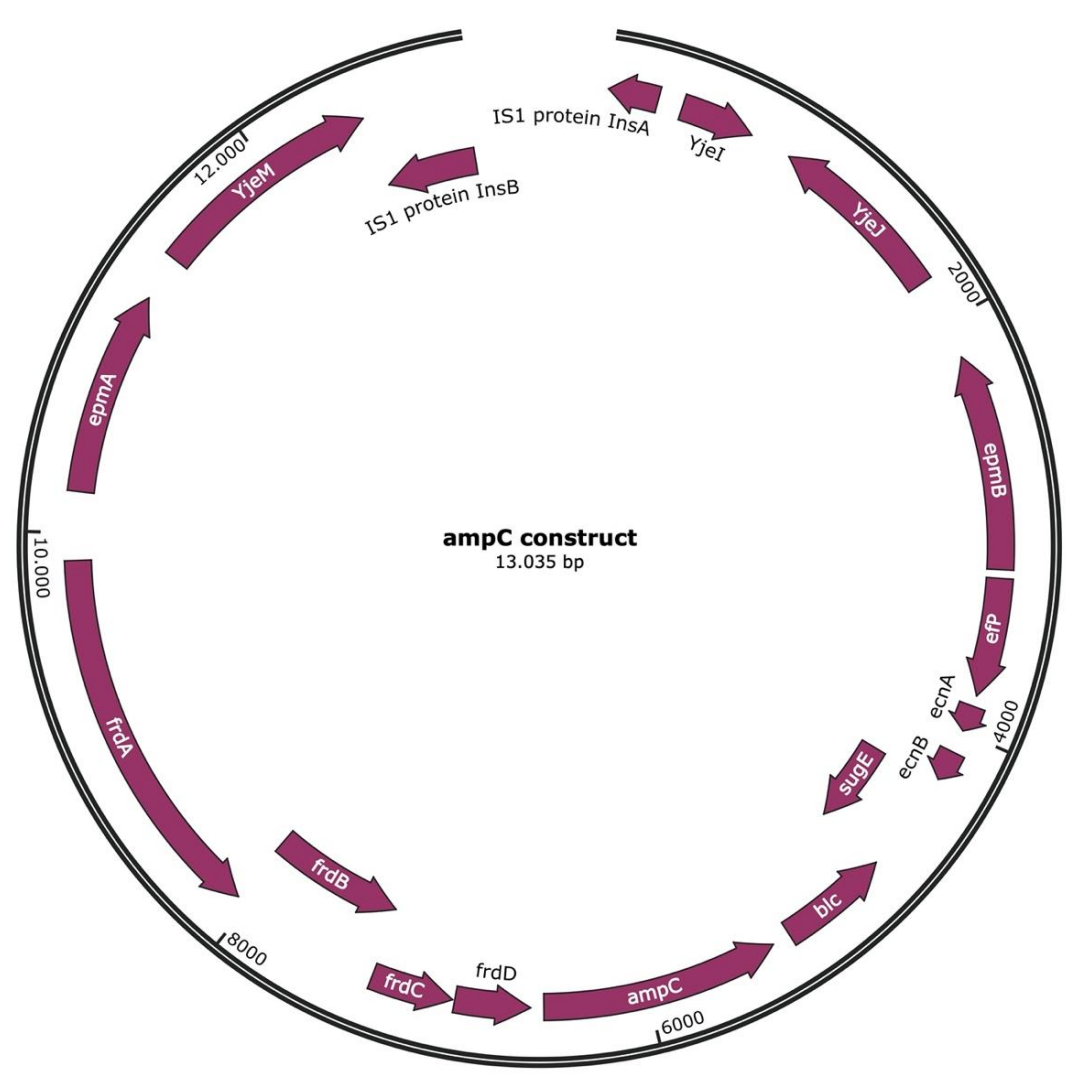
Annotated *ampC* transposon as isolated in eight out of nine evolved *E. coli* strains (endpoints with MIC for amoxicillin at 1024 µg/mL).

**Figure 4:**
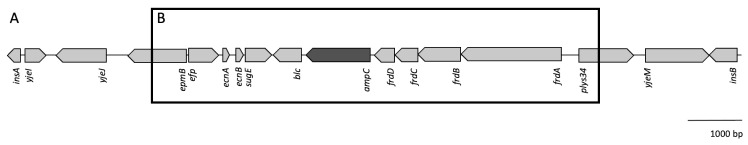
Sequence of the isolated band and *ampC* transposon as found in eight replicates in two different variations. Seven replicates (R2–R8) contained full sequence A, and one replicate contained sequence B (R9). The *ampC* gene is highlighted in dark grey.

DNA segments with the *ampC* gene were replicated as observed before in similar experiments, inducing de novo amoxicillin resistance in *E. coli* [9]. Plasmid isolation was performed in order to elucidate whether the replicated fragments remained part of the chromosome or were separated from genomic DNA. In all nine strains, the plasmid purification procedure yielded a band upon gel electrophoresis of the samples (Figure 2), even though the wild-type MG1655 that had been used as a starting point does not contain plasmids. The absence of plasmids was confirmed by sequencing the DNA of the wild-type strain and performing a search for all known plasmid types using PlasmidFinder 2.1 [19]. This procedure yielded no positive hits. DNA sequence analysis of the purified samples showed a contig highly enriched in *ampC* compared to the unpurified DNA samples. As usual in plasmid isolations, low amounts of genomic DNA were also present. Since the enriched DNA was originally derived from the genomic DNA, this could cause confusion. Hence, an additional purification step was introduced by cutting the bands out of the gel. Analysis of these latter samples confirmed that seven of the nine strains with induced resistance contained an identical 13,035-bp contig that contained *ampC* and had the insertion elements *insA* and *insB*, forming together IS*1*, at both ends (Figure 3 and Figure 4A). The presence of IS1 suggests that this contig can be called a transposable element or transposon. This transposon can be circular, or two or more repeats could be connected. One strain had the same transposon, but because the DNA was more fragmented, the assembly could only be performed by using the other transposons as a matrix. After this reconstruction, the transposon was identical to the other seven. The last strain contained a shorter, 7803-bp (Figure 4B) transposon that was identical to the transposons of the other strains but shortened at both ends. 

The size of the bands in Figure 2 is larger than the expected 13 kb. Possibly, the transposons formed complexes of several 13-kb units. Sequence analysis was, however, very unambiguous. The 13,035-bp unit was the only DNA present in the band cut out of the gel and the most dominant by far in the samples that were sequenced without this extra purification step. An earlier attempt with a different method to isolate plasmid DNA had yielded gels with similar bands but also smears, indicating contamination with chromosomal DNA. Given that each sample yielded only the identical 13 kb transposons and no other sequences, we are convinced that these are not experimental artifacts.

The transposon isolated from eight of the nine strains with induced amoxicillin resistance includes, most importantly from the point of view of resistance, the *ampC* gene (Figure 3). In addition, there are a fumarate reductase cluster consisting of four subunits (*frd*), lipocalin *blc*, the small multi-drug resistance gene *sugE*, the lipoproteins entericidin A and B (*ecnA* and *B*), the translation elongation factor P (*efp*), the lysine 2,3-aminomutase *epmB* and translation elongation factor plys34 (*epmA*). The longer fraction additionally includes two uncharacterized proteins, *yjeI* and *yjeJ*, as well as the inner membrane transporter *yjeM* and the IS*1* elements *insA* and *insB*. The same sequence is found within the genome of wild-type *E. coli* MG1655, as seen in Figure 4, but now also includes IS*1*, as indicated by the presence of *insA* and *insB*. *InsA* and *insB* flank the transposon in such a way that indicates it has been replicated from the genome as is usual for IS1. 

Comparison of the *ampC* promoter/attenuator region of the nine replicates with induced amoxicillin resistance and that of wild-type *E. coli* MG1655 showed minor differences, all of which, with the exception of one deletion and one insertion, were substitutions (Table 1). Seven out of eight resistant strains had a mutation in the promoter region of *ampC* at position −32. An additional point mutation was observed in two of the nine strains in the attenuator region of *ampC* at position +32. The strain with the short transposon had a mutation in the attenuator region of *ampC* at position +23. The transposons of the latter three strains that had extra *ampC* mutations also contained mutations in the *frdA* gene: a single point mutation in one strain, three point mutations in another strain with one mutation also situated in *frdB*, and a deletion in the third strain, all in different positions within the *frdA* gene. Finally, one strain had an additional mutation in *ecnA*, another one had two point mutations close to *epmB*, and a third one showed mutations closely before *yjeM*. 

### 2.2. Transfer of the ampC Transposon

The discovery of a DNA fragment resembling in some respects a transposon led to the question whether this could be transferable between *E. coli* cells. To examine this possibility, the nine resistant strains were mixed individually in the absence of antibiotics with equal cell numbers of a chloramphenicol-resistant recipient strain. This recipient also contained the *YFP* gene and was used before in similar experiments measuring rates of plasmid transfer [20,21]. The transformants were identified by their double resistance to amoxicillin and chloramphenicol. The term “transformants” was used since the cells and the transposon did not harbor genes or phages that could aid in conjugation or transduction. Therefore, the most likely method of transfer was transformation. The mating procedure was designed to mimic natural conditions, so to determine whether such events could happen in the natural environment of *E. coli*. To rule out induced chloramphenicol resistance in a non-transformed amoxicillin-resistant cell, the presence of the *YFP* gene was checked by measuring fluorescence to verify that a colony was truly a transformant. Only three of the nine strains with de novo amoxicillin resistance yielded some transformants, even though all potential donor strains possessed the *ampC*-containing transposon. The maximum number in the whole series of experiments was 0.12 transformants per million cells per hour, which is low compared to plasmid-containing cells, but still enough for a transformed resistant strain to emerge. Several transformants were randomly selected for isolation and analysis of the transferred transposon. The presence of *insAB* suggested that the transposon was taken up in its entirety from the donor strain. The two sequenced transposons isolated from transconjugants had minor mutations, but still showed an almost 100% identity with the transposon found in the donor strain, including the presence of *insA* and *insB*. Both had the same point mutation in *insB*, and one had a small insertion of 13 bps in *insA*. 

## 3. Discussion

The outcome of this study indicates that: (1) *E. coli*, when made resistant to amoxicillin by adapting it to stepwise increasing concentrations, forms a transposon that contains the *ampC* gene; (2) this transposon can be transferred to another fully susceptible *E. coli* strain, which then becomes resistant at the same level; (3) parts of the transposon are found regularly in certain types of beta-lactam resistance plasmids. 

The induction of almost completely identical *ampC* transposons by building up amoxicillin resistance indicates that the formation of these transposons is under strict control of the cellular machinery. The ninth transposon was a shortened version of the other eight. Similar parts of the genome were multiplied between 20 and 600 times in comparable experiments inducing amoxicillin resistance [3]. A highly comparable replication of the *ampC* gene can also be facilitated by a transducing phage [15]. The transposon was cut and multiplied with the help of the small and active transposable element IS*1* [22]. The element encodes the two out-of-phase open reading frames *insA* and *B’-insB*, which are necessary for its transposition. The *ampC* transposon together with IS*1* form a combined transposon. This transposon has in the process undergone some mutations. The exact mechanism of multiplication for this transposon is yet unknown. However, multiple methods of amplification or duplication are known for these types of transposable elements, including for IS*1*, by means of homologous recombination, which could apply here as well [23]. Transposons have been shown to form extra chromosomal structures and circles in yeast [24]. Some insertion sequences also form “mini-plasmids” or circular IS elements outside of the genome, such as IS*3* and IS*1* [25,26]. The circular step was mostly believed to be an intermediate step towards integration into a chromosome or a plasmid. In this case, the “mini-plasmid” of IS*1* could have taken up the *ampC* transposon and remained extra-chromosomal or fused back into the chromosome.

The increase of amoxicillin resistance correlated with the multiplication of the *ampC* gene and a mutation in the promoter region of *ampC* at position −32. Many different mutations are known to influence the activity of *ampC* [6], especially mutations in the promoter and attenuator region [7,12]. These findings were confirmed in this study. Specifically, the −32 promoter mutation has been shown to increase transcription initiation strongly by a factor between 8 and 46 [10,11,13]. Multiple mutations in the attenuator region, such as the +32 and +23 mutations found in this study, have also shown to be involved in an increase in *ampC* gene expression by reducing the stability of the hairpin structure [12]. 

A transposon can be transferred to other cells. This event is rare in comparison to the transfer rates of ESBL-plasmids [20], but in the same range as the transfer of tetracycline resistance plasmids [21]. Possibly, the transfer happens through transformation of the free-floating *ampC* transposon released from dead cells, due to the lack of conjugation genes or phages present to help induce conjugation or transduction. Transposon and other transposable elements can be transferred through natural transformation [27]. During transformation, some mutations were induced in the transposon, but only within *insA* and *insB*. Transfer could possibly also have taken place with the use of nanotubes, as described by Baidya et al., 2020 [28]. Many bacteria including *E. coli* harbor genes to produce nanotubes that can penetrate the walls of recipient cells. The tubes can be used to transport various molecules from cell to cell.

**Figure 5:**
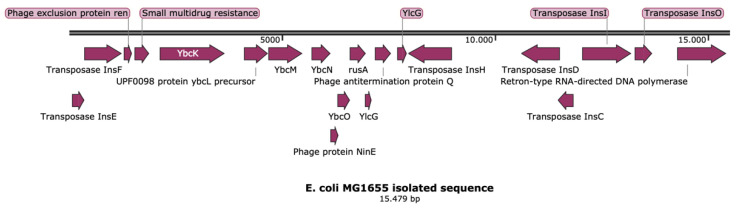
Isolated sequence using Qiagen plasmid maxi kit from wild-type *E. coli* MG1655.

Some transposable elements are induced as a result of stress [29,30]. Specifically, exposure to ampicillin increased the mobility of IS*1* and IS*5* from 0% to 17% in *E. coli* strains [31]. These elements, however, can also be found enriched without the cell being under stress, as indicated by the other transposable elements isolated from wild-type *E. coli*, shown in Figure 5. In this case, the stress response triggered the increase of specific transposable elements, which started cutting and forming fragments. The genesis of eight almost completely identical transposons in nine replicates indicates that the process was targeted rather than random. However, it cannot be excluded that other transposable elements could also have been triggered that did not result in any beneficiary changes. 

The *ampC* transposon induced by exposure to amoxicillin shows some resemblance with known beta-lactam resistance plasmids that were discovered in *E. coli* (Figure 4). In a certain way, the transposon could be considered as a precursor before incorporation in plasmids, with some genes lost in the process. An AmpC type beta-lactamase in plasmids that has been named after its discovery of high resistance to cephamycin is CMY [32]. CMY types have been shown to originate from *Citrobacter freundii* by transferring their chromosomal *ampC* gene to plasmids [4]. Some of the genes adjacent to *ampC* in our transposon in *E. coli* are also located in the genetic environment of CMY types [6]. In several types of plasmids, only the adjacent genes, *blc* and *sugE*, have been conserved, for example, with CMY-2 [18,33]. Similarly, the genetic environment of CMY-13 also contains the conserved genes *frdD*, *frdC*, and part of *frdB* [6]. In *E. coli*, these parts of the plasmids can be incorporated from a transposon after it was taken up or they could originate from the genome of the host cell with the help of IS*1* or other mobile elements, in a similar fashion as has been shown for *C. freundii* [4]. Other insertion sequences such as IS*Ecp1* can integrate within a plasmid with only the essential genes, as shown for extended-spectrum beta-lactamase-type CTX-M [34,35]. IS*Ecp1* has also shown to facilitate the integration of CMY-2 from the chromosome into a plasmid [36]. This mechanism can explain the expanding list of *ampC* variations and closely related genes. The availability of the amplified transposon combined with the genetic fine-tuning of the *ampC* gene makes it more effective against beta-lactam antimicrobials. After the transposon has been taken up by the cell or the host cell has obtained a plasmid, the *ampC* gene can be incorporated into plasmids and subsequently be transferred to other cells at higher rates. 

## 4. Material and Methods

### 4.1. Bacterial Strains, Evolution Experiment, and MICs

*Escherichia coli* MG1655 was used as starting strain for evolution experiments. Cells were grown at 37 °C in a defined minimal mineral medium containing 55 mM glucose with a pH of 6.9 and a buffer of 15.6 g/L of Na2H2PO4 [37]. This medium was chosen because it is better defined than complex media, and hence physiological measurements are more reproducible. Stock solutions of amoxicillin (10 mg/mL) were filter-sterilized and stored at 4 °C for a maximum of 2 weeks. In total, 9 replicate strains were exposed to stepwise increasing concentrations of amoxicillin as described before [38]. The starting concentration (1 µg/mL) was chosen as a quarter of the wild-type MIC (4 µg/mL) for amoxicillin. Every replicate was exposed to the starting concentration of amoxicillin, and a reference culture was grown without antibiotics. Cells were grown for 24 h at 37 °C and shaken at 200 rpm. When after 24 h the OD_600_ of the exposed culture was at least 75% of the OD_600_ of the reference culture, the population was considered adapted, and the antibiotic concentration was doubled in the subsequent incubation, with the flasks with lower concentration now serving as the reference culture for the flasks with doubled amoxicillin concentration. The experiment was continued until all replicates grew well at a final amoxicillin concentration of 1024 µg/mL. During the entire run of the evolution experiment, a control strain was grown without antibiotics. At the end of the evolution experiments, glycerol stocks of each replicate were stored at −80 °C.

MICs were measured every 3 to 4 days as described by Schuurmans et al. (2009) [39] in 96-well plates in a ThermoScientific Multiskan FC (Thermo Fisher Scientific’s, Breda, Netherlands) spectrophotometer plate reader. Plates were shaken and kept at 37 °C in a final volume of 150 μL with a starting OD_595_ of 0.05 for 23 h. Antibiotic concentrations increasing by a factor of 2 and ranging from 1 µg/mL to 2048 μg/mL were used. The lowest concentrations that limited the final OD to 0.2 or less was reported as the MIC.

To ascertain whether the induced resistance was permanent, the 9 replicates were separated into 3 groups of 3 replicates each. All groups were grown with daily transfers for 14 more days after reaching the endpoint. Group 1 was grown without antibiotics; group 2 was grown with a subinhibitory concentration for the wildtype of 4 µg/mL of amoxicillin, and group 3 was grown at 1024 µg/mL. The MICs were determined at the end of the 2 weeks.

### 4.2. Transfer Experiments

The ability of *E. coli* to transfer induced AmpC-mediated resistance by conjugation was tested in so-called transfer experiments. The 9 replicate endpoints of the evolution experiment with a MIC of 1024 µg/mL were used as donor strains, and a modified *E. coli* MG1655 strain with YFP and chloramphenicol resistance (kindly provided by MB Elowitz [40]) was used as the acceptor strain. Two methods were applied to increase the chance of finding transformants. In the first, 1-day-old cultures of the donor and acceptor strains were mixed together in a 1:1 ratio with an OD_600_ of 1 in minimal medium or in LB medium (5 g/L of NaCl (Sigma-Aldrich, Steinheim, Germany), 2.5 g of yeast extract (Scharlau-Microbiology, Barcelona, Spain), 5 g of bactotryptone (Brunschwig chemie, Amsterdam, The Netherlands). After 1 h, 24 h, and 48 h of co-incubation in flasks with shaking, the total numbers of acceptor, donor and transformant cells were determined by plating appropriate dilutions on antibiotic-selective LB agar plates (Scharlau-Microbiology, Barcelona, Spain). The antibiotic concentrations of the plates were 64 µg/mL of chloramphenicol to select for the acceptor strain, 64 µg/mL of amoxicillin for the donor strain, and 64 µg/mL of both amoxicillin and chloramphenicol to select the transformants. The second method consisted in mixing 50 µL of overnight cultures of both the donor and the acceptor strains in a 1:1 ratio and plating these directly onto selective plates. Transformant colonies on the double-antibiotic-selective plates were isolated for storage, and the MIC was determined for both amoxicillin and chloramphenicol. To verify whether transformants were truly transformants and not amoxicillin-resistant strains with rapidly acquired chloramphenicol resistance, colonies were tested for the YFP signal under a fluorescent microscope. Transformation was considered proven when the strain had increased resistance to both antibiotics compared to the wild-type, as well as a YFP signal.

### 4.3. DNA Isolation, Sequencing, and Data Analysis

All 9 endpoints, the wild-type strain, and 12 randomly selected transformants were subjected to plasmid isolation using a Qiagen Plasmid Maxi Kit (Qiagen Benelux B.V., Venlo, The Netherlands). Cell pellets collected from 400 mL of an overnight culture were used as substrate for the kit. The samples obtained were checked for purity with Nanodrop. If samples had a low concentration or were contaminated with salt, they were purified by ethanol precipitation. Samples of the 9 endpoints were further purified by cutting the single band that was obtained from a 0.7% agarose gel and extracting the DNA from the gel by dissolving the gel and reconcentrating the DNA. Sequencing was performed by BaseClear B.V. (Leiden, The Netherlands), utilizing the Illumina NovaSeq 6000 system. De novo assembly was performed using the generated short reads. The quality of Illumina reads was improved using the error correction tool BayesHammer [41]. Error-corrected reads were assembled into contigs using SPAdes version 3.10 [42]. The order of contigs and the distances between them were estimated using the insert size information derived from an alignment of the paired end reads to the draft assembly. Consequently, contigs were linked together and placed into scaffolds using SSPACE version 2.3 [43]. Using Illumina reads, gapped regions within scaffolds were (partially) closed using GapFiller version 1.10 [44]. Finally, assembly errors and nucleotide disagreements between the Illumina reads and scaffold sequences were corrected using Pilon version 1.21 [45]. Initially, 1 sample was sequenced and assembled using PacBio for long reads and separately by Illumina for short reads. The two methods gave the exact same results. Illumina alone was therefore considered adequate and used for the other replicates. As a control for the purified band, the full extractions with the Qiagen plasmid maxi kit were also sequenced without any further purification.

The obtained scaffold sequences were annotated using RAST 2.0 [46]. The annotated sequences were analyzed using Snapgene viewer 5.1.7 (from insightful Science; available at snapgene.com) and CLC genomics workbench 21 (https://digitalinsights.qiagen.com/, Accessed on 1 February 2021). CLC workbench was used to perform further reference-based assembly where needed and reference mapping to reads to determine whether a duplication of the ampC transposon was present in the samples. Both BLAST (https://blast.ncbi.nlm.nih.gov/Blast.cgi Accessed on 8 December 2020) and CLC workbench were used to determine mutations between the ampC transposon of the strains and the similar genomic environment of wild-type *E. coli* MG1655. The reference wild-type genome with accession number U00096.3 was downloaded from the NCBI database (https://www.ncbi.nlm.nih.gov/genbank/ Accessed on 12 March 2021). 

## 5. Conclusions

Plasmids are ubiquitously found in bacterial cells, but their origin and evolution are rarely known. The outcome of this study shows that resistance can be induced by exposure to the beta-lactam antibiotic amoxicillin in *E. coli* by multiplication and mutation of the chromosomal *ampC* gene. This results in transposons that are infrequently transferred to other cells, most likely through transformation. The transposons show similarities with gene clusters found regularly in beta-lactam resistance plasmids. This in turn strongly suggests that plasmids can be generated de novo by transformation, incorporation, and/or genetic rearrangement of such transposons with other genetic elements of bacteria, including the chromosome. These observations offer an explanation for the large diversity of plasmids found in nature and identify processes by which the dynamics of plasmids are maintained.

## Figures and Tables

**Table 1:** Mutations found per replicate (for endpoints after evolution) and transformant strains (R5-6 and R5-10). All mutations are single point mutations, unless specifically addressed.

Replicate/Strain	Mutations in Promoter/Attenuator Region *ampC*	Mutations in Other Genes
2	−32, +32	*frdA*
3	−32	*ecnA*
4	−32, +32	3× *frdA*, 1× *frdB*
5	−32	
6	−32	1× before *epmB*, 1× *epmB*
7	−32	
8	−32	2× before *yjeM*
9	−32, +23	Deletion in *frdA*
R5-6	−32	*insB*
R5-10	−32	*insB*, insertion in *insA*

NB A formatted version of the table is attached after the References.

## Data Availability

Sequence data have been submitted to GenBank, but an accession number was not yet available at the moment of printing. All data can be obtained from the corresponding author by E-mail request.
